# Patterning effects of FGF17 and cAMP on generation of dopaminergic progenitors for cell replacement therapy in Parkinson’s disease

**DOI:** 10.1093/stmcls/sxaf004

**Published:** 2025-03-12

**Authors:** Amalie Holm Nygaard, Alrik L Schörling, Zehra Abay-Nørgaard, Erno Hänninen, Yuan Li, Adrian Ramón Santonja, Gaurav Singh Rathore, Alison Salvador, Charlotte Rusimbi, Katrine Bech Lauritzen, Yu Zhang, Agnete Kirkeby

**Affiliations:** Novo Nordisk Foundation Center for Stem Cell Medicine (reNEW), Department of Biomedical Sciences, Faculty of Health and Medical Sciences, University of Copenhagen, 2200 Copenhagen, Denmark; Novo Nordisk Foundation Center for Stem Cell Medicine (reNEW), Department of Biomedical Sciences, Faculty of Health and Medical Sciences, University of Copenhagen, 2200 Copenhagen, Denmark; Department of Experimental Medical Sciences, Wallenberg Center for Molecular Medicine (WCMM) and Lund Stem Cell Center, Lund University, SE-221 84 Lund, Sweden; Novo Nordisk Foundation Center for Stem Cell Medicine (reNEW), Department of Biomedical Sciences, Faculty of Health and Medical Sciences, University of Copenhagen, 2200 Copenhagen, Denmark; Novo Nordisk Foundation Center for Stem Cell Medicine (reNEW), Department of Biomedical Sciences, Faculty of Health and Medical Sciences, University of Copenhagen, 2200 Copenhagen, Denmark; Department of Experimental Medical Sciences, Wallenberg Center for Molecular Medicine (WCMM) and Lund Stem Cell Center, Lund University, SE-221 84 Lund, Sweden; Department of Neuroscience, Faculty of Health and Medical Sciences, University of Copenhagen, 2200 Copenhagen, Denmark; Novo Nordisk Foundation Center for Stem Cell Medicine (reNEW), Department of Biomedical Sciences, Faculty of Health and Medical Sciences, University of Copenhagen, 2200 Copenhagen, Denmark; Novo Nordisk Foundation Center for Stem Cell Medicine (reNEW), Department of Biomedical Sciences, Faculty of Health and Medical Sciences, University of Copenhagen, 2200 Copenhagen, Denmark; Department of Neuroscience, Faculty of Health and Medical Sciences, University of Copenhagen, 2200 Copenhagen, Denmark; Novo Nordisk Foundation Center for Stem Cell Medicine (reNEW), Department of Biomedical Sciences, Faculty of Health and Medical Sciences, University of Copenhagen, 2200 Copenhagen, Denmark; Department of Experimental Medical Sciences, Wallenberg Center for Molecular Medicine (WCMM) and Lund Stem Cell Center, Lund University, SE-221 84 Lund, Sweden; Novo Nordisk Foundation Center for Stem Cell Medicine (reNEW), Department of Biomedical Sciences, Faculty of Health and Medical Sciences, University of Copenhagen, 2200 Copenhagen, Denmark; Department of Experimental Medical Sciences, Wallenberg Center for Molecular Medicine (WCMM) and Lund Stem Cell Center, Lund University, SE-221 84 Lund, Sweden; Department of Neuroscience, Faculty of Health and Medical Sciences, University of Copenhagen, 2200 Copenhagen, Denmark

**Keywords:** FGF17, dopaminergic progenitors, Parkinson’s disease, Midbrain-hindbrain boundary, human pluripotent stem cell differentiation, transplantation

## Abstract

Cell replacement therapies using human pluripotent stem cell-derived ventral midbrain (VM) dopaminergic (DA) progenitors are currently in clinical trials for treatment of Parkinson’s disease (PD). Recapitulating developmental patterning cues, such as fibroblast growth factor 8 (FGF8), secreted at the midbrain-hindbrain boundary (MHB), is critical for the in vitro production of authentic VM DA progenitors. Here, we explored the application of alternative MHB-secreted FGF-family members, FGF17 and FGF18, for VM DA progenitor patterning. We show that while FGF17 and FGF18 both recapitulated VM DA progenitor patterning events, FGF17 induced expression of key VM DA progenitor markers at higher levels than FGF8 and transplanted FGF17-patterned progenitors fully reversed motor deficits in a rat PD model. Early activation of the cAMP pathway mimicked FGF17-induced patterning, although strong cAMP activation came at the expense of EN1 expression. In summary, we identified FGF17 as a promising alternative FGF candidate for robust VM DA progenitor patterning.

## Graphical Abstract

**Figure:**
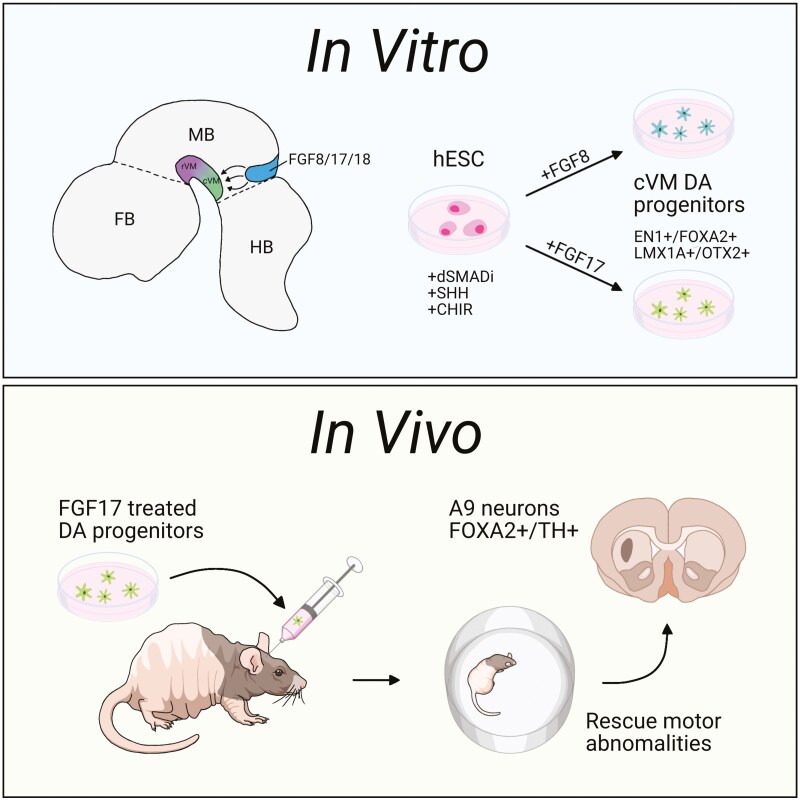
In vitro and in vivo effects of FGF17

## Introduction

Parkinson’s disease (PD) is an incurable neurodegenerative disorder, the motor symptoms of which are caused by a relatively selective loss of dopamine (DA) neurons in the substantia nigra. Engraftment of VM DA progenitors to the putamen to replace the lost endogenous cells is a promising restorative therapeutic strategy to ensure continuous DA release at physiological levels,^1^ and several approaches are currently in clinical trial.^2^ The differentiation protocols used to generate VM DA progenitors from human pluripotent stem cells (hPSCs) have been improved by adjusting early patterning with growth factors.^3-6^ Although the current clinically applied differentiation protocols efficiently yield relatively high-purity VM DA progenitor cells^3,7-9^ the release criteria are still only based on 1-2 VM markers, and further fine-tuning to increase purity and reproducibility is crucial to ensure the success of serial large-scale GMP manufacturing for clinical use.

The isthmic organizer is a key signaling center located at the midbrain-hindbrain boundary (MHB) of the developing vertebrate embryo.^10,11^ It plays a fundamental role in establishing positional information and regional identity of the midbrain and hindbrain. In particular, the expression of WNT1 and members of the fibroblast growth factor 8 (FGF8) family are crucial for the function of this secondary organizer in guiding the patterning of the VM in model organisms.^12^ Based on this, FGF8 is applied in several protocols for manufacturing hPSC-derived VM DA progenitors, where it functions to induce expression of the VM DA progenitor marker Engrailed 1 (EN1).^3,5,6^ High levels of EN1 and LMX1A expression are important for the in vitro generation of *bona fide* caudal VM DA progenitors and correlate with a good graft outcome in animal models of PD.^13,14^ While in vitro protocols have focused only on the use of FGF8, previous studies have shown that not only FGF8, but also FGF17 and FGF18 are expressed at the MHB in chick and mouse.^15^ FGF8, FGF17, and FGF18 belong to the same canonical subfamily (FGF8 subfamily), acting on the same receptors,^16^ and ectopic application of these FGFs has been shown to stimulate expansion of the midbrain in the developing mouse.^15^ Furthermore, FGF17 has been found to be not only more highly expressed in the developing midbrain region but also for longer periods of time when compared to both FGF8 and FGF18.^17^ In humans, however, the dynamics of MHB FGF expression have not been thoroughly explored.

Here, we show that FGF17 is expressed more strongly than FGF8 in the human MHB and that FGF17-mediated patterning of hPSCs yielded VM DA progenitors with significantly higher expression of *FOXA2* and *LMX1A* when compared to FGF8. Transplantation of FGF17-derived VM DA progenitors rescued motor deficits in rats and produced DA-rich and highly innervating grafts. Through scRNAseq, we uncovered increased cyclic adenosine monophosphate (cAMP) signaling as an underlying mechanism for the increased LMX1A induction in FGF17-treated cells. In summary, we have identified FGF17 as an alternative and developmentally relevant candidate for efficient VM DA patterning in vitro.

## Materials and methods

### Cell culturing

Mycoplasma-free, karyotyped RC17 (Roslin cells) hESCs were cultured on Laminin-521 (1 µg/cm^2^) coated plates. RC17 was cultured in StemMACS iPS Brew XF (Miltenyi Biotec) and passaging was performed using 0.5 mM EDTA dissociation at 37°C. For the first 24 hours after passaging 10 µM ROCK inhibitor (Miltenyi Biotec) was included in the media. Specification of hESC toward VM fates was performed in accordance with the Nolbrant et al protocol.^6^ Adaptation to the current protocol was the addition of either FGF8b (100 ng/mL, #423-F8-025/CF), FGF18 (100 ng/mL, #8988-F18-050) or FGF17b (100 ng/mL, #319-FG-025) to test for optimal VM DA patterning. FGFs were in all cases sourced from R&D Systems to avoid any comparability issues related to the vendor. Isoform b was chosen for FGF17 due to its potency and analogy to FGF8b. FGF18 does not undergo alternative splicing. When testing the synergistic effect of FGF8b and FGF17b only 50 ng/mL of each was added to compare a total of 100 ng/mL FGF to the respective controls. Cells were harvested at days 16 and 42 for analysis.

For the additional factor screening experiments VM patterned cells were treated with PDE8 inhibitor PF-04957325 300 nM (MedChem, #HY-15426), dibutyryl-cAMP 500 µM (Sigma-Aldrich, #D0627), PDGFR inhibitor CP673451 100 nM (R&D Systems, #CP 673451 5993/10), PDGFC 1 µg/mL (R&D Systems, #1687-CC-025) or NT-3 10 or 20 ng/mL (R&D Systems, #267-N3-005) on top of FGF8b 100 ng/mL from days 9 to 16. Analysis was performed at days 16 and 42. In experiments with ERK inhibition cells were treated with Trametinib 50 nM (Biotechne #7709/10) day 9-16 on top of FGF8b 100 ng/mL from days 9 to 16. Analysis was performed on day 16.

### Animal experiments

All procedures were conducted in accordance with the European Union Directive (2010/63/EU) and were approved by the local ethical committee at Lund University and the Swedish Department for Agriculture (ethical permit number 5.2.18-10992/18). Adult, female Sprague Dawley (SD) and athymic, nude rats (Hsd:RH-*Foxn1*^rnu^) were purchased from Charles River and Envigo, respectively, and were housed on a 12:12-hour light:dark cycle with ad libitum access to food and water, *n* = 4 SD rats, *n* = 7 nude rats. For all surgical procedures, rats weighting >225 g and/or older than 3 months were anesthetized via intraperitoneal (IP) injection of a 20:1 or 3:2 mixture of fentanyl-Dormitor or ketaminol-Dormitor (Apoteksbolaget), respectively, according to the weight. The DA neurons of the rats were unilaterally ablated by intracranial injection of 10.5 µg 6-hydroxydopamine (6-OHDA) to the medial forebrain bundle (MFB). The extent of the lesion was assessed by an amphetamine-induced rotations test. For this purpose, 3.5 mg/kg amphetamine was administered by IP injection, and mean net turns per min were measured over 90 minutes, following a 10 minutes staggered start. Cryopreserved VM DA progenitors derived from RC17 hESCs were prepared as previously described.^6,18^ 3e5 cells were unilaterally transplanted to SD- and nude rat striatum as described previously.^19^ SD rats were injected at the coordinates AP, +0.8; ML, −2.8; DV, −4.5, and nude rats at the coordinates AP, + 0.9/+1.4; ML, −3.0/−2.6; DV, −5.0/−4.0. SD rats were administered 10 mg/kg cyclosporine by daily IP injections 2 days prior to transplantation, and until euthanization, 18 weeks post-transplantation.

### Immunolabeling

Brains were fixed in 4% (wt/vol) paraformaldehyde by perfusion, according to standard protocol. The brains were removed, and post-fixed over-night before dehydration in 25% (wt/vol) sucrose. Brains were sectioned with a thickness of 35 µm in 1:8 series by using a freezing microtome (Leica), and were stored in *Buffer A* (13 mM NaH_2_PO_4_, 38 mM Na_2_HPO_4_ 30% (vol/vol) ethylene glycol, 30% (vol/vol) glycerol) at −20°C. Immunolabeling was performed on free-floating sections, using the Corning Netwells system (Merck). The sections were placed on a slowly rotating (~100 rpm) orbital shaker during all incubations, which were done at room temperature, unless specified otherwise. Sections were washed for 3 × 5 minutes in PBS between all steps unless otherwise specified. For antigen retrieval, sections were incubated in *Buffer B* (10 mM Tris Base, 1 mM EDTA Solution, 0.05% Tween 20, pH 9.0, H_2_O) at 80°C for 30 minutes. During immunohistochemistry—where the detection system was horseradish peroxidase (HRP)-based—endogenous peroxidases were inactivated by incubation in *Buffer C* (10% (vol/vol) methanol, 3% (vol/vol) H_2_O_2_, PBS) for 30 minutes. If necessary to decrease background toning in IHC, sections were first blocked in *Buffer D* (2.5% vol/vol Triton-X, 5% (vol/vol) species-specific serum), supplemented with avidin and biotin, using the Avidin/Biotin Blocking Kit (Vector Laboratories) according to the manufacturer’s instructions. Sections were blocked in *Buffer D* for 1 hour. The sections were after this incubated with one or several of the primary antibodies ALDH1A1 (1:1000, AbCam, ab52492), hNCAM (1:1000, Santa Cruz Biotechnology, Sc-106), TH (1:2000, Merck Millipore, AB152), TH (1:1000, Merck Millipore, AB1542), HuNu (1:1000, Merck Millipore, MAB1281), FOXA2 (1:1000, Santa Cruz Biotechnology, sc-101060) and LMX1A (1:1000, Merck Millipore, AB10533) over night. The sections were then blocked for 15 minutes in *Buffer D*. After this, the section was incubated in the biotinylated secondary antibody or fluorescently labeled secondary antibodies for 1 hour for IHC and immunofluorescence (IF), respectively. For IHC, the sections were thereafter incubated in ABC HRP or ABC alkaline phosphatase complex according to the manufacturer’s instructions (Vector Laboratories). The chromogenic substrate development in IHC was thereafter performed by incubating the sections in the enzymatic system-compatible substrate solution until the desired color intensity had developed. The following substrates were used: (i) 0.5 mg/mL DAB, catalyzed by the addition of 0.125% (vol/vol) H_2_O_2_, (ii) Vector Blue or (iii) Vector® DAB, with or without supplementation with nickel (Vector Laboratories), prepared according to the instructions. Sections were then immediately washed several times briefly in PBS to avoid color saturation. If necessary during IHC, counterstaining was done using mild progressive labeling with hematoxylin. Chromagenically labeled sections were mounted and dehydrated by incubation in step-wise-increasing concentrations of ethanol and were then cleared. Lastly, the sections were coverslipped in an appropriate mounting media. Cells were fluorescently labeled with primary and secondary antibodies (Supplementary Table S3), as previously described.^6^

### Imaging and quantification

The rat brains were processed and sectioned in series of 8, and one series was used for the quantifications. For quantification of TH^+^ cells in DAB-labeled grafts, brightfield, Z-stack images under 20× magnification were captured and stitched automatically by using a Leica DMI6000B with the LAS-X software program. TH^+^ cells in FGF17 grafts were counted manually in the software program Fiji (version 2.1.0) whereas the TH^+^ cells in the STEM-PD graft were counted manually at the microscope. The quantifications of TH^+^ cells are presented as the estimated total number of TH^+^ cells per 1e5 cells transplanted per animal using the formula:


$$\begin{array}{l} Sum\;\;\;TH+cells=\frac{\underset{per\;\;\;animal}{\sum }TH+\left(cells\right)}{3e5\;\;\;\left(cells\right)}x\;\;\;8\;\;\;\left(series\right) \end{array}$$

Overview, 10× brightfield images of the chromagenically labeled rat brains were acquired on an Axio Scan Z1 (Zeiss) microscope. IF images were acquired using the 40× objective on the confocal microscope Stellaris (Leica). Brightfield and IF images were processed in the software program QuPath (version 0.4.3). Imaging of immunolabeled cells was performed on the Leica AF600 widefield epifluorescence microscope, using Leica LAS-X software. Images were processed with Fiji (version 2.1.0).

### Quantitative RT-PCR

RNA was collected on days 16 and 42 from 500 000 cells. For RNA isolation the RNeasy Micro Plus kit (Qiagen) was used. cDNA synthesis was performed from 1 µg purified RNA using the Maxima First strand synthesis kit (ThermoFisher, #K1642). SYBR green (Roche, #04887352001), primer mix (fwd + rev, **Supplementary Table S4**), and cDNA for each qRT-PCR reaction were pipetted using the liquid dispensing robot iDOT (Dispendix). Ct values were measured over 40 cycles on the Lightcycler 480 (Roche). Each reaction was run in duplicates and the average Ct value was used for calculating fold change values over hESCs by the 2^−∆∆Ct^ method.^20^

### Flow cytometry analysis

For intracellular flow cytometry, the cells were diluted to 1.0 million cells/mL in *Buffer X* (1% (vol/vol) N-2™ supplement, CTS™ Neurobasal™ Medium). LIVE/DEAD™ Fixable Violet or Near-IR dye (Thermo Fisher) or dimethyl sulfoxide (DMSO) was added, and the samples were incubated for 15 min at room-temperature. After a wash in *Buffer Y* (1% (vol/vol) bovine serum albumin (BSA), DPBS (-Ca^2+^/-Mg^2+^)), cells were fixed and permeabilized by the addition of 1X Fix/Perm buffer (BD Biosciences), prepared according to the manufacturer’s instructions. After two sequential washes in 1X Perm/Wash buffer (BD Biosciences), prepared as instructed, cells were stored at 4°C overnight, protected from light. Cells were then incubated with FOXA2-APC (Cat#130-124-043, 1:80) and OTX2-VioB515 (Cat#130-121-202, 1:320) antibodies in 1X Perm/Wash buffer for 30 min at 4°C. Cells were washed once in 1x Perm/Wash buffer, and twice in *Buffer Y*. The compensation beads Anti-REA beads (Miltenyi Biotec) were used for FOXA2-APC and OTX2-VioB515, ArC-reactive beads (Miltenyi Biotec) for Live/Dead Fixable Violet and Near-IR Dyes, all prepared as recommended. Cells were interrogated on a BD LSRFortessa™ using fully stained dorsal forebrain or ventral hindbrain progenitor cells as a biological negative control to set the FOXA2 and OTX2 gates. The data was analysed with FlowJo

### Single-cell RNA sequencing

Four batches of paired FGF8- and FGF17-treated day 16 VM DA progenitors (8 samples total) were thawed in wash media containing DMEM/F12 + 5%KOSR. Next, cells were counted, spun down, and resuspended in PBS(wo Ca and Mg) with 0.5%BSA at 5mio cells per ml. 0.5mio cells per sample were stained with 0.5 µg of unique cell surface hashing antibody (Biolegend) and incubated for 30 minutes at 4°C. After antibody tagging, cells were washed 3 times in PBS(wo Ca and Mg) with 0.5%BSA and resuspended at 1000 cells/µl in PBS(wo Ca and Mg) + 0.5%BSA. The eight samples were prepared to be run across two 10× sequencing lanes by pooling two batches of paired FGF8 and FGF17 samples at 1:1:1:1 ratio for both sequencing lanes. Each sequencing lane was loaded with 25 000 cells. For sequencing of our day 16 VM DA progenitors, we combined 10X scRNA-seq with CITE-Seq for sample multiplexing to sequence 8 different replicates across 2 protocols.^21^ We performed cell surface hashing on 4 different batches per treatment group and each 10× lane ran 2x FGF8 and 2 xFGF17 treated groups to correct for batch effect. The raw sequence data has been preprocessed with the Alevin-fry pipeline^22^ and the downstream analyses were performed with Seurat v.4.1.0. The acquired count matrices were filtered to remove low-quality cells and doublets. All mitochondrial and ribosomal genes were removed from the dataset before continuing with normalization by SCTransform. The dimensionality of the data was reduced with PCA and UMAP, followed by graph-based clustering and annotations were based on cluster-wise differentially expressed gene identification using the FindAllMarkers function from Seurat v.4.10. The top 5 genes with the highest *avg_log2FC* from each cluster were selected for heatmap visualization. Differentially expressed gene analysis between the FGF8- and FGF17-treated cells was performed using DESeq2 v.1.30.1^23^ on all cells as implemented in the FindMarkers Seurat function. Genes with *P_val_adj* < .05 were accepted as significantly differentially expressed genes. To compare the signaling network between the 2 studied protocols, we analyzed the datasets for the 2 protocols separately with identical parameters. We first identified regulons (modules of genes that are targeted by transcription factors) and quantified regulon activity in each cell using Pyscenic v.0.11.2.^24^ We then used Domino v.0.1.1^25^ to infer the signaling networks. Transcription factors that were specific to each cluster were identified using the Mann-Whitney *U* test, and top transcription factors (up to 10) with *P*-values below .001 were selected. We next identified potential receptors that activated transcription factors, where receptors and transcription factors were considered significantly connected if Pearson correlation >0.23 with a maximum of 10 receptors for each transcription factor. Afterward, we acquired the ligand signaling partners for each connected receptor from the CellphoneDB2 database. Ligands that were not expressed were excluded. With the top 10 transcription factors identified for each cluster and their connected receptors and ligands, we generated the cluster-specific top regulon activity/transcription factor activation heatmap, the transcription factor and receptor correlation heatmap, the global signaling network, as well as the global intercellular signaling network.

### Bulk RNAseq

RC17 cells were patterned toward VM according to Nolbrant et al^6^. On day 9, cells were treated with either FGF8b 100 ng/mL or FGF17 100 ng/mL for 15 minutes, 1 hour, 4 hours, and 24 hours. Upon sample collection cells were washed once with cold PBS −/− and lysed in RLT buffer (QIAGEN). RNA was extracted using RNeasy Mini kit (QIAGEN) including on-column DNase treatment (QIAGEN). RNA quality was assessed using the HS RNA screentape kit (Agilent) on Tapestation 4200 (Agilent) following the manufacturers protocol. Samples with RIN^e^ values >8 were included for the next steps. Libraries were prepared using the NEBNext Poly(A) mRNA magnetic isolation module (NEB #E7490S) in combination with NEBNext Ultra II directional RNA library prep kit for Illumina (NEB #E7765) following the manufacturer’s protocol. Libraries were eluted in 17.5 µl 0.1× TE after the final clean up. Library yields were quantified using the Qubit and quality control was performed on Tapestation 4200 (Agilent) using an HS D1000 screentape kit (Agilent) following the manufacturer’s protocol. Libraries were diluted to 10 nM and pooled. One additional cleanup was performed on the pooled sample to eliminate an adaptor contamination in one sample. All 24 libraries were sequenced together on one flow cell on Illumina NextSeq 2000. The obtained sequencing data underwent quality control and alignment with the nf-core/rnaseq (v3.10.1)^26^ pipeline. Default parameters were used, except for setting skip_trimming to true, and hg38 was used as the reference genome for the alignment. To ensure the data and alignment quality, we inspected the MultiQC report from the pipeline. The uncorrected gene count matrix generated by the pipeline was quality-filtered by removing genes with low counts across samples. The resulting count matrix served as an input for differential expression analysis, performed using the DESeq2 package (v1.40.2),^27^ to identify genes with differential expression patterns between FGF8 and FGF17 treatments at different time points of stimulation. Genes with *P_val_adj* < .01 and *log2FoldChange ± *0.4 were considered as differentially expressed.

### Spatial gene expression visualization

Publicly available spatial transcriptomics data from post-conception week 5 human neural tube^28^ was used to visualize spatial gene expression in the developing human brain. The spatial data were converted into an AnnData object, a format required by the Tangram^29^ package. To create the figures, we modified the plotting functions from Tangram to suit our purposes.

### Statistical analysis

Statistical tests and visualization were done by using R (version 4.2.2) or Prism (10.0.02), with a critical significance level of alpha ≤0.05. Equality of variance was investigated with Brown-Forsythe tests and normality with Shapiro-Wilk tests and manual inspection of frequency distributions. Homoskedastic and normal data was analyzed with the omnibus test analysis of variance (ANOVA), and data not meeting these criteria with a Kruskal-Wallis test. Post-hoc analysis was done by using Dunnet’s multiple-comparison test. Paired *t*-test analysis was applied in cases where each biological replicate experiment (ie, each differentiation batch) was performed with parallel testing of different conditions on the same batch of differentiated cells. Data points originating from the same biological differentiation batch were treated as paired data points in the *t*-test. Comparison of two groups with normal distributions was done with paired or non-paired 2-sided *t*-tests, with consideration for equality of variance. Unless otherwise specified, the data is expressed as mean ± SEM.

## Results and discussion

### Additional FGF8 subfamily members induce VM patterning

**Figure 1.**
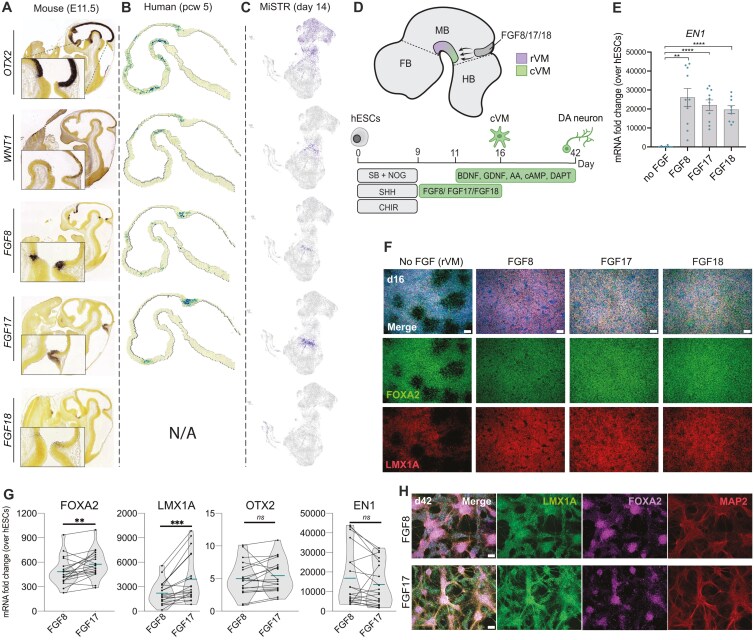
FGF subfamily members are capable of ventral midbrain induction. A. Expression of key MHB genes in the E11.5 mouse embryo, from the Allen Brain Atlas.^30^ B. Spatial transcriptomic data of MHB genes in the developing human postconceptional week (pcw) 5.5 fetus.^28^ C. ScRNAseq data from an in vitro human neural tube model (MiSTR) at d14 (16). D. Schematic of FGF8 subfamily members expressed at the MHB, involved in the induction of caudal (cVM) vs rostral (rVM) VM patterning, and schematic of the in vitro protocol used for hESC differentiation. E. mRNA expression of EN1 in d16 VM progenitors treated with FGFs from d9-16. Analyzed by Brown-Forsythe ANOVA followed by Dunnet’s multiple comparisons. *P < .05, **P < 0.01, ***P < 0.001, ****P < 0.0001, ns: non-significant, *n* = 8 (no FGF), *n* = 10 (FGF8, FGF17), *n* = 9 (FGF18). F. Immunolabeling of FOXA2 and LMX1A in d16 hESC-derived VM DA progenitors, patterned with FGFs from d9-16. G. Paired analysis of mRNA expression of key VM DA progenitor markers at d16, comparing FGF8 and FGF17. Data points from 1E (EN1, *n* = 10) are also included in these graphs. **P < 0.01, ***P < 0.001, EN1 *P* = 0.11, ns: non-significant, analyzed with a paired *t*-test, *n* = 19. H. Immunolabeling of FOXA2, LMX1A, and MAP2 in d42 hESC-derived DA neurons. All scalebars: 100 μM.

To assess the most relevant FGFs at the MHB, we first revisited available data from the developing mouse and human embryos. Investigation of expression patterns in the developing mouse brain (E11.5),^30^ shows that *fgf17* is more broadly expressed around the MHB than *fgf8*, while *fgf18* is only weakly expressed (Figure 1A). A similar broad expression pattern of *FGF17* at the MHB junction was observed in a recently published spatial transcriptomic data of human postconceptional week 5 (pcw 5) fetal tissue^28^ (Figure 1B). In line with this, we have previously found *FGF17* to be expressed at the MHB of an in vitro model of the developing human neural tube, and here *FGF17* showed both broader and stronger expression than *FGF8* (Figure 1C).^31^ While data is not available for *FGF18* in the human embryo at pcw 5, we found *FGF18* to be very weakly expressed in our in vitro neural tube model at day 14 (d14), aligning with a weak signal in the mouse at E11.5 (Figure 1A-C).

To compare the ability of different MHB-associated FGFs from the FGF8 subfamily to fine-tune hPSC-derived VM DA progenitors, we added either FGF8b (from here on referred to as FGF8), FGF17, or FGF18 from day (d) 9-16 of a published directed differentiation protocol for the production of clinical-grade VM DA progenitors,^6,7^ and differentiated the cells in parallel (Figure 1D). By qRT-PCR analysis we found that all 3 FGFs induced a significant increase in caudal VM DA marker *EN1* mRNA expression levels (Figure 1E) while maintaining cultures with a high purity of FOXA2+/LMX1A + progenitors (Figure 1F). Through qRT-PCR analysis of parallel batches of VM differentiations, we found that FGF17 induced significantly higher expression of *FOXA2* and *LMX1A* compared to FGF8, while *OTX2* and *EN1* expression did not differ (Figure 1G). We found no difference in the expression of these markers between FGF17- and FGF18-patterned batches (Supplementary Figure S1A). Given the higher in vivo relevance of *FGF17* over *FGF18* at the MHB (Figure 1A-C) we therefore decided to focus on the actions of FGF17 for the remainder of the study. Nuclear flow cytometry of FGF17-patterned d16 VM DA progenitor cells showed unchanged proportions of FOXA2/OTX2 double-positive cells compared to paired FGF8-patterned cells (Supplementary Figure S1B), indicating that the positive effect of FGF17 on FOXA2 expression may be due to increased expression levels within each cell. When combining FGF8 and FGF17 in the same differentiation (50% dose of each), we found no further beneficial synergistic effect compared to FGF8 alone (Supplementary Figure S1C-E). This is in line with the fact that FGF8 and FGF17 bind to the same FGF receptor,^16^ whereby synergy between the 2 factors is not expected. To assess the maturation capacity of FGF17-treated VM DA progenitors, we performed in vitro maturation, and found that both FGF17- and FGF8-patterned VM DA progenitors produced cultures of neurons expressing TH, MAP2, FOXA2, and LMX1A at d42 of differentiation (Figure 1H and Supplementary Figure S1F). Analysis of the mature cultures by qRT-PCR on d42 however did not show detectable differences in the levels of mature DA neuron markers (ie, LMX1A, EN1, TH, NURR1, Supplementary Figure S1G) between the FGF8- and FGF17-patterned cultures.

### FGF17-patterned VM DA progenitors reverse motor deficits in a rat model of PD

**Figure 2.**
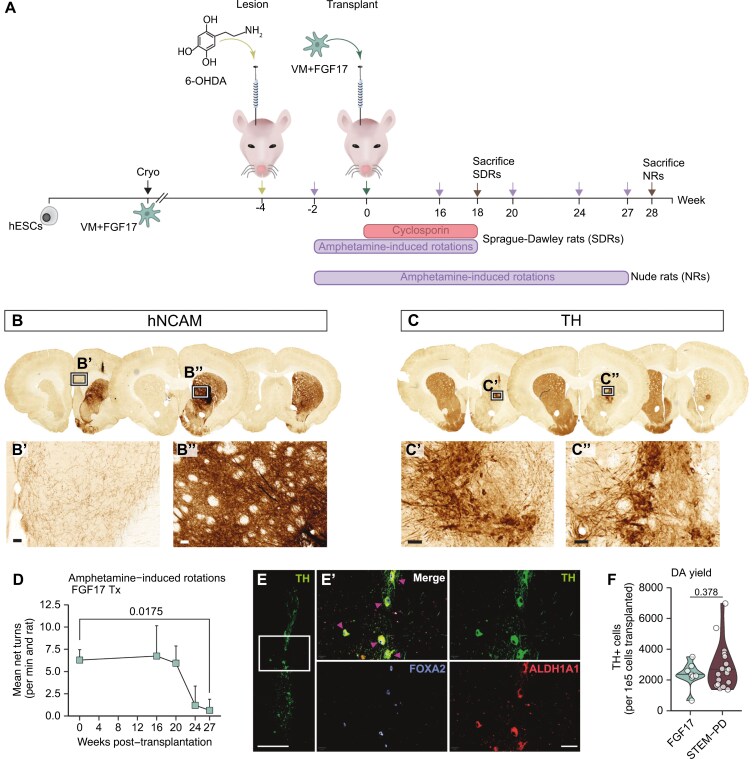
Functionality of FGF17-patterned DA neurons upon transplantation to Parkinsonian rats. A. Nude rats (NRs) and Sprague-Dawley rats (SDRs) were unilaterally lesioned with 6-OHDA and then transplanted with cryopreserved d16 FGF17-patterned cells. The functionality of the transplanted cells was evaluated at 16-, 20-, 24-, and 27-week post-transplantation through amphetamine-induced rotations. SDRs were immunosuppressed with Cyclosporin upon transplantation. B-C. Representative images of immunohistochemical stainings for hNCAM (B) and TH (C) in transplanted rat brains, showing dense human fiber innervation and TH-rich grafts, scalebars = 50 µM. D. Mean net turns per min in amphetamine-induced rotation test from rats transplanted with FGF17-patterned cells. *n* = 7 (week 0), *n* = 4 (week 16, 20, 24), *n* = 3 (week 27). A Welch *t*-test was used to determine differences between weeks 0 and 27 (*P*-value = 0.0175). E. Representative images of FGF17 graft at 27 weeks, stained for TH, FOXA2, and ALDH1A1. Triple-positive cells are indicated by triangles. Overview scale bar = 200 μm, zoom-in scalebar = 40 μm. F. Quantified total yield of TH + cells per 1e5 cells transplanted in FGF17 grafts at 27 weeks compared to the clinical cell product STEM-PD (STEM-PD data obtained from Kirkeby et al., 2023). *n* = 8 (FGF17), *n* = 18 (STEM-PD). Statistical significance determined by *t*-test, *P* = 0.378.

To ascertain whether the FGF17-patterned VM DA progenitors were also functional upon engraftment, FGF17-patterned VM DA progenitors were transplanted to the striatum of unilaterally, 6-hydroxydopamine (6-OHDA)-lesioned Sprague-Dawley or nude rats (Figure 2A). Immunohistochemical assessment showed dense human neural fiber innervation of the rat striatum (Figure 2B) and DA neuron-rich grafts (Figure 2C). The transplanted cells yielded full amelioration of motor deficits at 27-weeks post-transplantation in the amphetamine-induced rotation test (Figure 2D). We next confirmed that the FGF17 grafts expressed markers of the A9-subtype of DA neurons through co-labeling of TH with the floorplate marker FOXA2^13,32^ and the A9 DA neuron marker ALDH1A1^33^ (Figure 2E). The FGF17-patterned grafts generated on average 2335 ± 812 mature TH^+^ neurons per 1e5 transplanted cells (mean ± SD). This is comparable to the yield obtained from the clinical-grade FGF8-patterned VM DA progenitor cell product STEM-PD, which is currently being tested in a European phase- I/IIa clinical trial for PD, ClinicalTrials.gov number NCT05635409 (2835 ± 1466 TH^+^ cells, Figure 2F, data from Kirkeby et al^7^).

### RNA-sequencing reveals differences in FGF8 and FGF17 signaling

**Figure 3.**
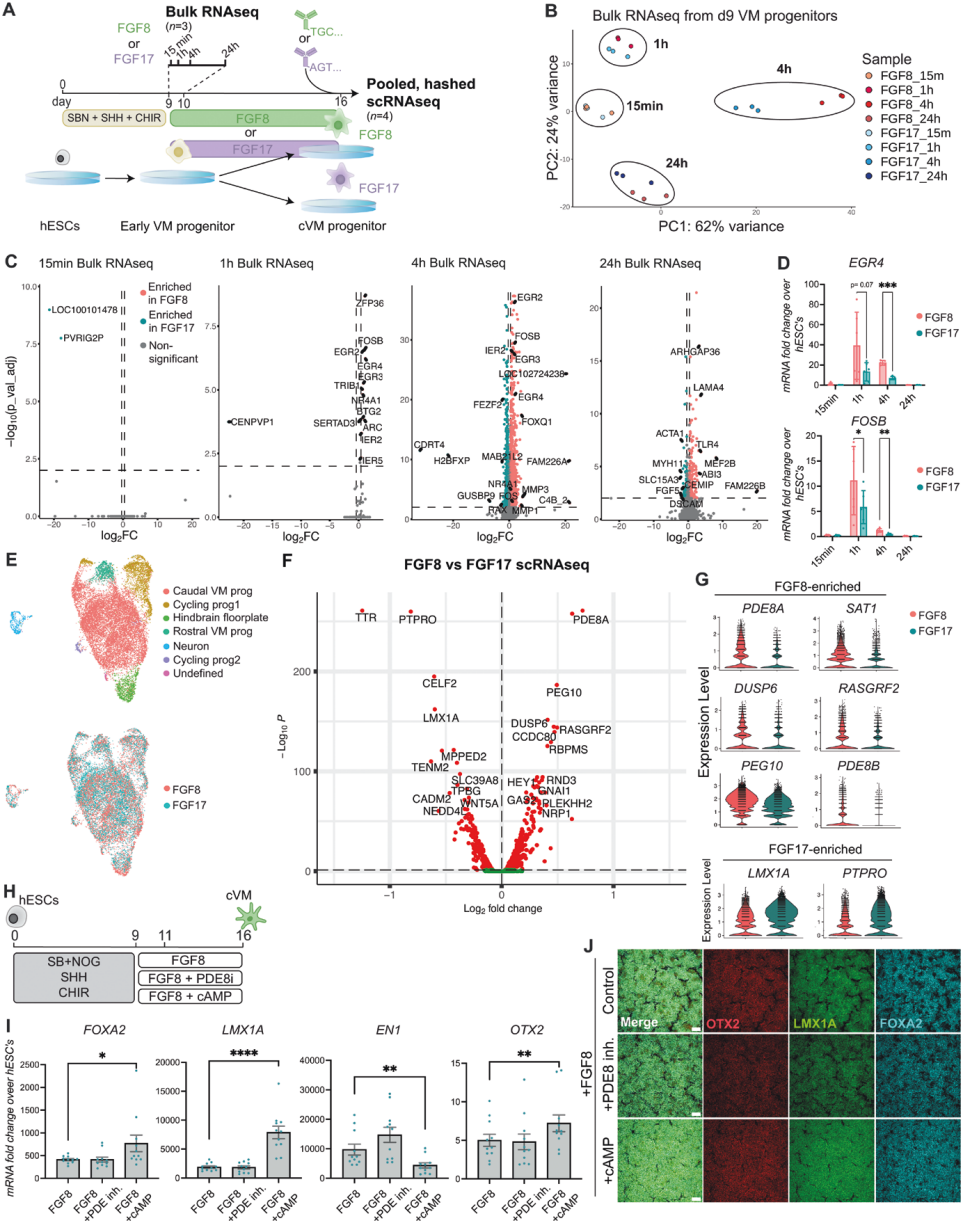
Addition of cAMP during VM patterning mimicks the effect of FGF17. A. Experimental outline for bulk and scRNAseq. FGF8- or FGF17-patterned d9 VM DA progenitors were (1) collected at 15 minutes, 1 hour, 4 hours, or 24 hours after FGF addition for bulk RNAseq (*n* = 3 per condition), or (2) differentiated until d16 for scRNAseq, *n* = 4. B. PCA plot comparing bulk RNAseq of FGF8 and FGF17 treated VM DA progenitors at all four timepoints, *n* = 3. C. Volcano plot of DEGs at 15 minutes, 1 hour, 4 hours, and 24 hours between FGF8- and FGF17-treated VM DA progenitors, cutoffs = *P*_val_adj < 0.01 and log2FoldChange ± 0.4. D. mRNA levels of early response genes EGR4 and FOSB after addition of FGFs. A paired *t*-test was used to test differences for each time point, *n* = 5-6. E. UMAP projection of scRNAseq data from FGF8 and FGF17 d16 VM DA progenitors. F. scRNAseq volcano plot of DEGs day 16, cutoffs = *P*_val_adj < 0.05 and log2FoldChange ± 0. Dots above the dashed line mark significantly expressed genes. G. Violin plots of top DEGs between FGF8 and FGF17 cells in the scRNAseq data. H. Outline of in vitro differentiation strategy to test target pathways uncovered by RNAseq. I. Quantitative RT-PCR of key VM markers in d16 progenitors treated with either PDE8i or cAMP together with FGF8 from days 9 to 16. One-way ANOVA for paired data followed by Dunnet’s multiple comparisons test: *P < .05, **P < .01, ***P < .001, ****P < .0001, ns: non-significant, *n* = 11. J. Immunocytochemistry showing co-staining of OTX2, LMX1A, and FOXA2 in d16 VM DA progenitors treated with PDE8i or cAMP, scalebar: 100 μM.

To uncover the biological pathways underlying the differences observed between FGF8- and FGF17-patterned cells, we performed 2 different RNA sequencing studies: (1) A time-course bulk RNAseq experiment on d9 VM progenitors treated with either FGF8 or FGF17 for up to 24 hours; and (2) A scRNAseq experiment on hashtag-labeled d16 VM DA progenitors patterned with either FGF8 or FGF17 for 7 days, from d9 to 16 (Figure 3A). For the bulk RNAseq, differences in FGF response pathways were investigated at the earliest time points immediately after initiation of FGF treatment (ie, at 15 minutes, 1 hour, 4 hours, and 24 hours after adding the FGFs, *n* = 3 for each FGF). Principal component analysis and differentially expressed gene (DEG) analysis showed that at 15 minutes post-FGF addition, there were negligible differences between the groups, but that the groups differed transcriptionally at the 1, 4, and 24-hour time-points (Figure 3B,C and Supplementary Table S1). The DEG analysis at 1-4 hours post-FGF treatment showed a strong induction of ERK/MEK-pathway associated early response genes, including FOSB, *EGR2, EGR3, EGR4*, and *IER2* (Figure 3C and Supplementary Figure S2A). Interestingly, these early response genes were found to be more upregulated in FGF8-treated cells compared to FGF17-treated cells (Figure 3C, Supplementary Figure S2A). This observation was confirmed by qRT-PCR in additional samples at 1 and 4 hours post-FGF treatment (Figure 3D).

Next, we performed scRNAseq on hashtag-labeled d16 VM DA progenitors patterned with either FGF8 or FGF17 (*n* = 4 differentiation experiments, each experiment testing FGF8 and FGF17 in parallel, Figure 3A). All 8 batches of differentiated cells showed similar transcriptional profiles at d16 (Supplementary Figure S2B,C), affirming the high similarity between VM batches differentiated with either FGF8 or FGF17. As expected, the majority of cells sequenced produced a large cluster of caudal VM DA progenitors marked by expression of *FOXA2*, *LMX1A*, *OTX2,* and *EN1* (Figure 3E, Supplementary Figure S2B,C). Both treatments also gave rise to a small proportion of rostral VM progenitors, marked by the absence of *EN1* and the presence of *OTX2, LMX1A,* and *FOXA2*. Furthermore, we found a small cluster of contaminating hindbrain floorplate progenitors (*FOXA2*^+^ but *OTX2*^-^) in both treatment groups, arising primarily from one of the 4th differentiation experiments, VM#4 (Figure 3E, Supplementary Figure S2C). Analysis of the top DEGs between all FGF8- and FGF17-treated cells revealed enrichment of *PDE8A*, *PDE8B,* and *DUSP6* in the FGF8-treated cells (Figure 3F,G and Supplementary Table S2). The PDE8 family members are phosphodiesterases that play a major role in the hydrolysis of cAMP and *DUSP6* (MKP3) is a protein phosphatase that has previously been found to be co-expressed with FGF8 at the MHB.^34^ FGF17-treated cells in contrast were confirmed to express higher levels of LMX1A and of protein tyrosine phosphatase receptor type O (PTPRO) (Figure 3F,G and Supplementary Figure S2C), which is a receptor that has recently been shown to be enriched on DA progenitor cells.^14^

To investigate the potential mechanism of action behind the differential VM patterning between FGF8- and FGF17-stimulated cells, we tested the addition of commercially available agonists and antagonists of the pathways identified in the bulk and scRNAseq analyses in combination with FGF8 treatment. Based on the increased ERK-related signaling observed in the FGF8-treated cells seen in the bulk RNAseq experiment (Figure 3C,D), we first tested an ERK inhibitor (ERKi) from d9-16 together with FGF8 treatment from d9-16. While we observed no effect on the expression of *FOXA2* and *LMX1A,* we found a significant decrease in *EN1* expression when ERKi was included in the differentiation from d9-16 (Supplementary Figure S3A). In addition, we found expression of the diencephalic marker *BARHL2* to be significantly upregulated in ERKi-treated cells, resembling the phenotype observed in the “No FGF” group (Supplementary Figure S3A). These results show that ERK activity is necessary for FGF8-mediated induction of *EN1* expression as well as repression of the diencephalic marker *BARHL2*, and that ERK inhibition does not recapitulate the FGF17 phenotype.

As the scRNAseq data further indicated the involvement of the cAMP pathway in the differential action between FGF17 and FGF8, we next proceeded to test the addition of a membrane-permeable cAMP (dibutyryl-cAMP, referred to as cAMP) as well as a PDE8 inhibitor (PF-04957325, PDE8i). The addition of cAMP is in most DA protocols used during the late stage of DA differentiation (after d16) to induce neuronal maturation.^4-6,35^ However, in these experiments, we tested cAMP as well as the PDE8i together with FGF8 earlier in the protocol, from d9-16. Surprisingly, the early addition of cAMP to FGF8-treated cultures caused a significant increase in the expression of VM markers *FOXA2*, *LMX1A, OTX2,* and *SHH* by d16 of differentiation (Figure 3I,J and Supplementary Figure S3B,C), thereby exacerbating the phenotype observed for FGF17- compared to FGF8-patterned progenitors (Figure 1F). This indicated that increased activation of the cAMP pathway may be a main mechanism differing between FGF8 and FGF17 signaling. However, early treatment with cAMP also, similar to inhibition of ERK, induced a significant decrease in expression of the caudal VM marker *EN1* as well as an upregulation of the diencephalic marker *BARHL2*, implying that the cells generated with early addition of cAMP were of a more rostral VM identity which is not beneficial for DA generation (Figure 3H and S3B,C). Interestingly, treatment with a PDE8i, which would also be expected to increase endogenous levels of cAMP, did not yield the same effects as cAMP in the VM DA progenitors (Figure 3I,J and Supplementary Figure S3B,C). This implies that the VM DA progenitors are not at this stage producing high levels of cAMP endogenously. By d42 of culture maturation we found no difference in mRNA levels of the DA markers *TH, NURR1, LMX1A,* or *EN1* indicating that the observed differences in progenitor patterning did not change the outcome of in vitro maturation (Supplementary Figure S3D).

Finally, to uncover more details on the signaling dynamics of FGF8 vs FGF17, we employed the computational tool Domino^25^ to identify intercellular signaling and transcription factor activation specifically in our “caudal VM prog” cluster (Supplementary Figure S3E**).** Among the most important networks expressed in the FGF8-treated caudal VM progenitor cluster were components of the TGF and PDGF pathway, governed by *EN1* and *FOXA1*. In the FGF17-treated cluster, signaling through the neurotrophin ligand-receptor *NTRK3* was predicted to be related to *LMX1A* expression. To investigate the potential role of these pathways we tested PDGFR inhibition or activation and the addition of neurotrophin 3 (NT-3) in combination with FGF8 treatment. However, by d16 of differentiation, we observed no significant difference in mRNA levels of key VM DA markers between the treatment groups, and concluded that these pathways were not crucial for VM DA patterning (Supplementary Figure S3F-H).

In summary, our data shows that the use of FGF17 might be beneficial compared to FGF8 for producing efficacious VM DA cells for transplantation to PD. We have previously shown that the addition of FGF to the cell differentiation protocol is crucial to ensure a high yield of caudal DA-producing VM progenitors over the rostrally adjacent subthalamic nucleus progenitors, which do not yield DA neurons upon maturation.^13^ However, the use of other FGF8 subfamily members in hPSC protocols remains largely neglected, despite their recognition at the MHB in model organisms decades ago. Studies from mouse and chick demonstrated that FGF8 and FGF17 could act synergistically during early MHB patterning,^17,36–38^ however, we observed no synergistic effect during in vitro patterning, and we hypothesize that the synergistic effect observed in vivo is likely due to differences in temporal and spatial expression levels between the 2 genes. Overall, our data indicates that FGF17 is the most highly expressed FGF in the human MHB and that both FGF17 and FGF18 can work redundantly and independently of FGF8 to govern caudal human VM DA patterning. This is in line with the knowledge that these 3 FGF8 subfamily ligands all function on the same receptors.^39^

Despite the similar patterning effects, minor differences in activated signaling pathways between FGF8 and FGF17 were evident. Although the protocols for patterning VM DA progenitors are already very efficient, even minor improvements to purity can be crucial for successful product release when manufacturing cells in a GMP setting for clinical use. The transcriptional differences between FGF8 and FGF17 resulted in the identification of the cAMP pathway as a pathway that, when activated early, can increase expression of VM genes LMX1A and FOXA2. However, this comes at the expense of the crucial VM DA progenitor marker EN1, which we have previously shown to be required for producing successful DA grafts.^13^ Interestingly, another VM DA progenitor product which is currently in clinical trial for PD by BlueRock Therapeutics includes cAMP in the early patterning of their VM DA progenitors, from d10-16.^4^ Based on this, it would be relevant to investigate if there is a minimal threshold of EN1 expression required at the progenitor stage for achievement of optimal in vivo efficacy. In summary, we demonstrate here FGF17 as an alternative member of the FGF8 subfamily with apparent similar efficiency as FGF8 at generating VM DA progenitors and neurons, and with a potential to induce a more robust transcriptional phenotype at the progenitor stage, with increased expression of key markers LMX1A and FOXA2.

## Supplementary Material

sxaf004_suppl_Supplementary_Materials

sxaf004_suppl_Supplementary_Table_S2

sxaf004_suppl_Supplementary_Tables_S1

## Data Availability

Spatial data from human 5 pcw fetal tissue is available at https://github.com/linnarsson-lab/developing-human-brain/ and can be accessed through https://doi.org/10.1126/science.adf1226. Developing mouse brain data is available from Allen Developing Mouse Brain Atlas, https://developingmouse.brain-map.org. Data from the in vitro model of the developing human neural tube (MiSTR) can be accessed through https://www.ncbi.nlm.nih.gov/geo/query/acc.cgi?acc=GSE135399. scRNAseq and bulk RNAseq datasets generated specifically for this study are accessible on https://www.ncbi.nlm.nih.gov/geo/query/acc.cgi?. acc=GSE266683 (bulk) and https://www.ncbi.nlm.nih.gov/geo/query/acc.cgi?acc=GSE266683 (scRNAseq). The code is available in the github repository https://github.com/kirkeby-lab/FGF8_FGF17
