# Supplementary material for: Patterning effects of FGF17 and cAMP on generation of dopaminergic progenitors for cell replacement therapy in Parkinson’s disease
Source: Stem Cells. 2025 Mar 12;43(3):sxaf004. doi: 10.1093/stmcls/sxaf004 (PMC11976395; doi:10.1093/stmcls/sxaf004)
Supplement: sxaf004_suppl_Supplementary_Materials [file sxaf004_suppl_supplementary_materials.pdf]

Supplementary figures and tables

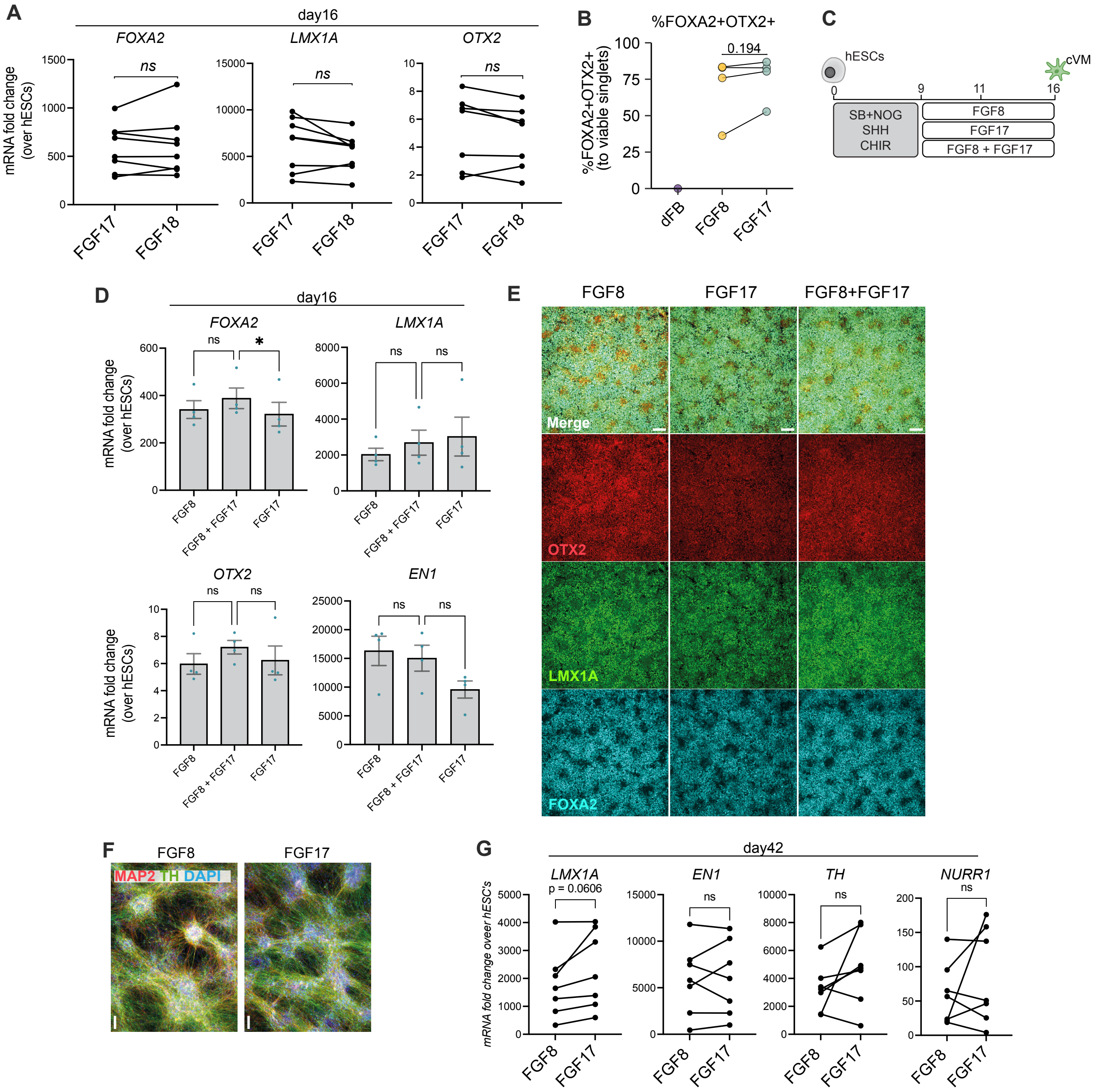

Figure S1. Comparison of FGF8- and FGF17-patterned VM DA progenitors

A. Comparison of mRNA expression of key VM markers in FGF17- and FGF18-treated day 16 progenitors analysed by a paired t-test. FGF17 data points are reused from figure 1G. ns: non-significant, n=7. **B.** Flow cytometric analysis of FOXA2/OTX2 double-positive cells. Paired t-test, p= 0.194, n=4. **C.** Experimental layout for testing the synergistic effect of FGF8 and FGF17. **D.** mRNAexpression of key VM markers in day 16 VM progenitors treated with FGF8, FGF17 or FGF8 and FGF17 together. Paired one-way ANOVA or Friedman test with Dunnet's or Friedmans multiple comparison's test for normal and non-normal distributed data, n =4. **E.** ICC of day16 cells treated with No FGF, FGF8, FGF17, or FGF8 and FGF17 combined, scalebar=100µM. **F.** Immuolabelling of day 42 FGF8- and FGF17-treated cells, scalebar = 100µM. **G.** mRNA expression of mature DA neuron markers in day42 cultures. A paired t-test was used to compare FGF8- and FGF17-treated cells, n= 7.

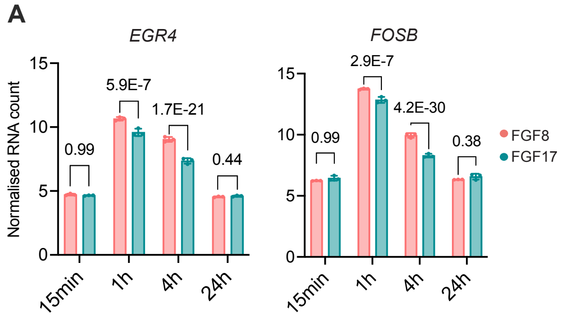

	EGR4, FGF8			EGR4, FGF17		
	Rep1	Rep2	Rep3	Rep1	Rep2	Rep3
15min	4,7	4,67	4,8	4,67	4,62	4,67
1h	10,78	10,68	10,5	9,92	9,46	9,4
4h	8,86	9,24	9,03	7,53	7,11	7,43
24h	4,58	4,56	4,51	4,62	4,65	4,55

	FOSB, FGF8			FOSB, FGF17		
	Rep1	Rep2	Rep3	Rep1	Rep2	Rep3
15min	6,25	6,26	6,22	6,36	6,3	6,67
1h	13,68	13,79	1376	13,11	12,63	12,81
4h	9,73	10,05	10,05	8,22	8,19	8,46
24h	6,32	6,34	6,34	6,29	6,68	6,73

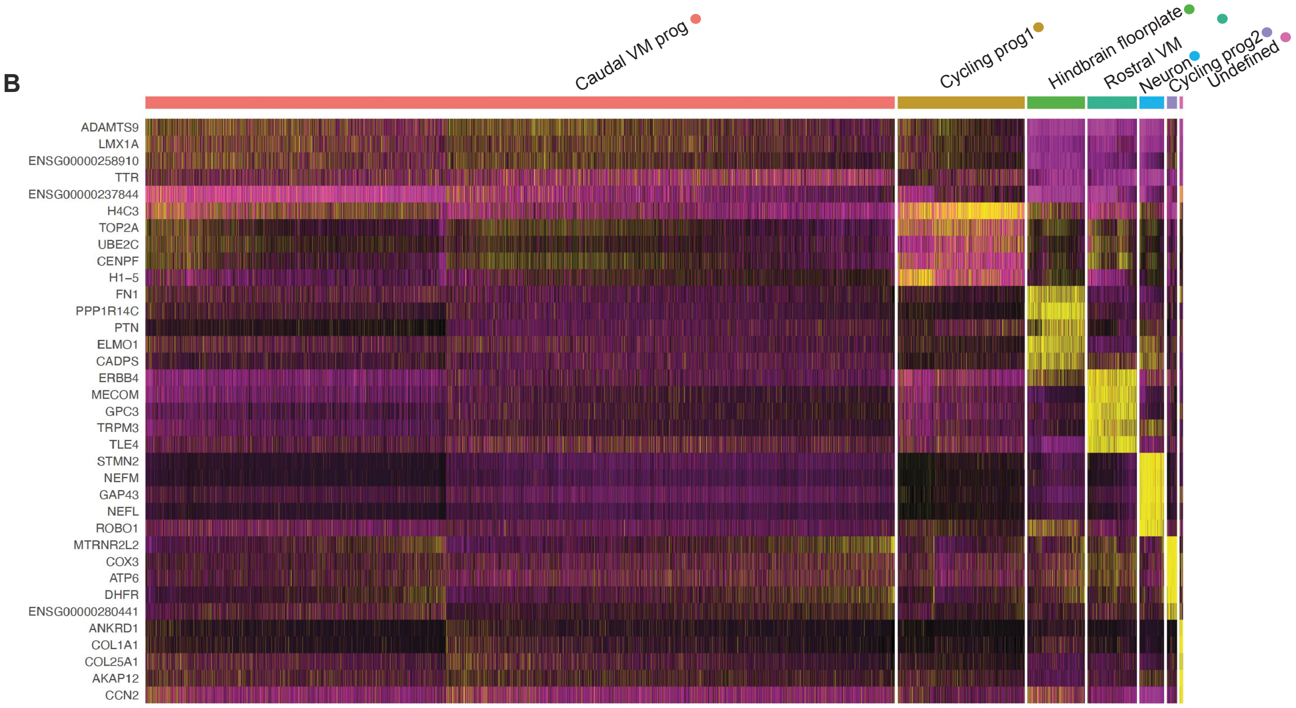

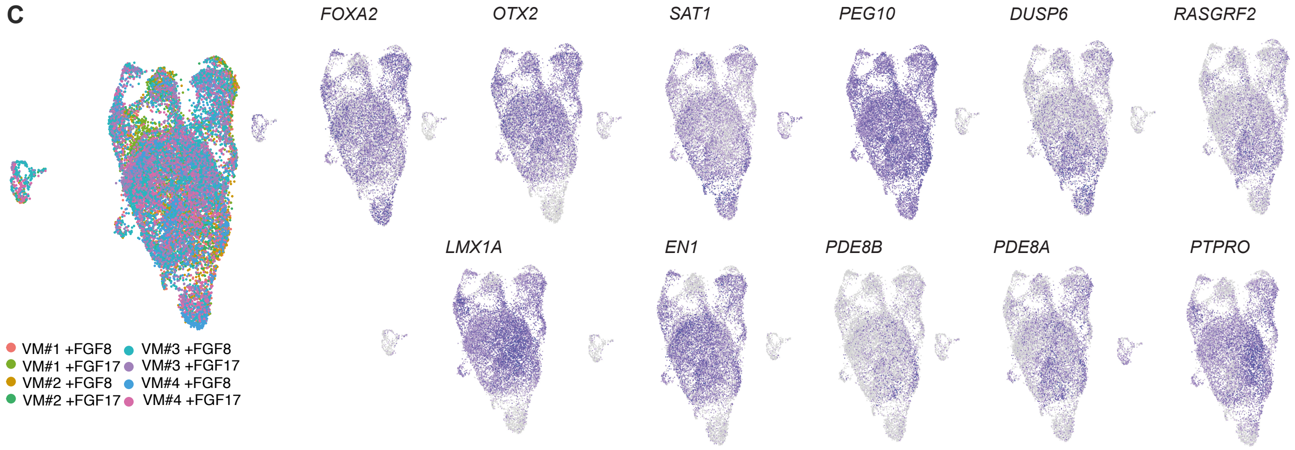

Figure S2. Gene expression in day 16 FGF8- and FGF17-patterned progenitors
A. Left: Bulk RNAseq results of FOSB and EGR4 RNA levels at 15min, 1hr, 4hr and 24hr post FGF treatment, n=3. Graph showing mean +/- SD and adjusted p-values from the DEG analysis. Right: Tables showing raw values of normalized RNA counts of FOSB and EGR4 from bulk RNAseq. **B.** Heatmap showing DEGs in each of the annotated clusters in the day 16 FGF8+FGF17 scRNAseq dataset. **C.** Feature plots in UMAP space for key VM genes and genes found to be differentially expressed between FGF8- and FGF17-patterned progenitors on day 16 (from Fig. 3E). Top left plot shows hashtags specific to each biological replicate included in the experiment.

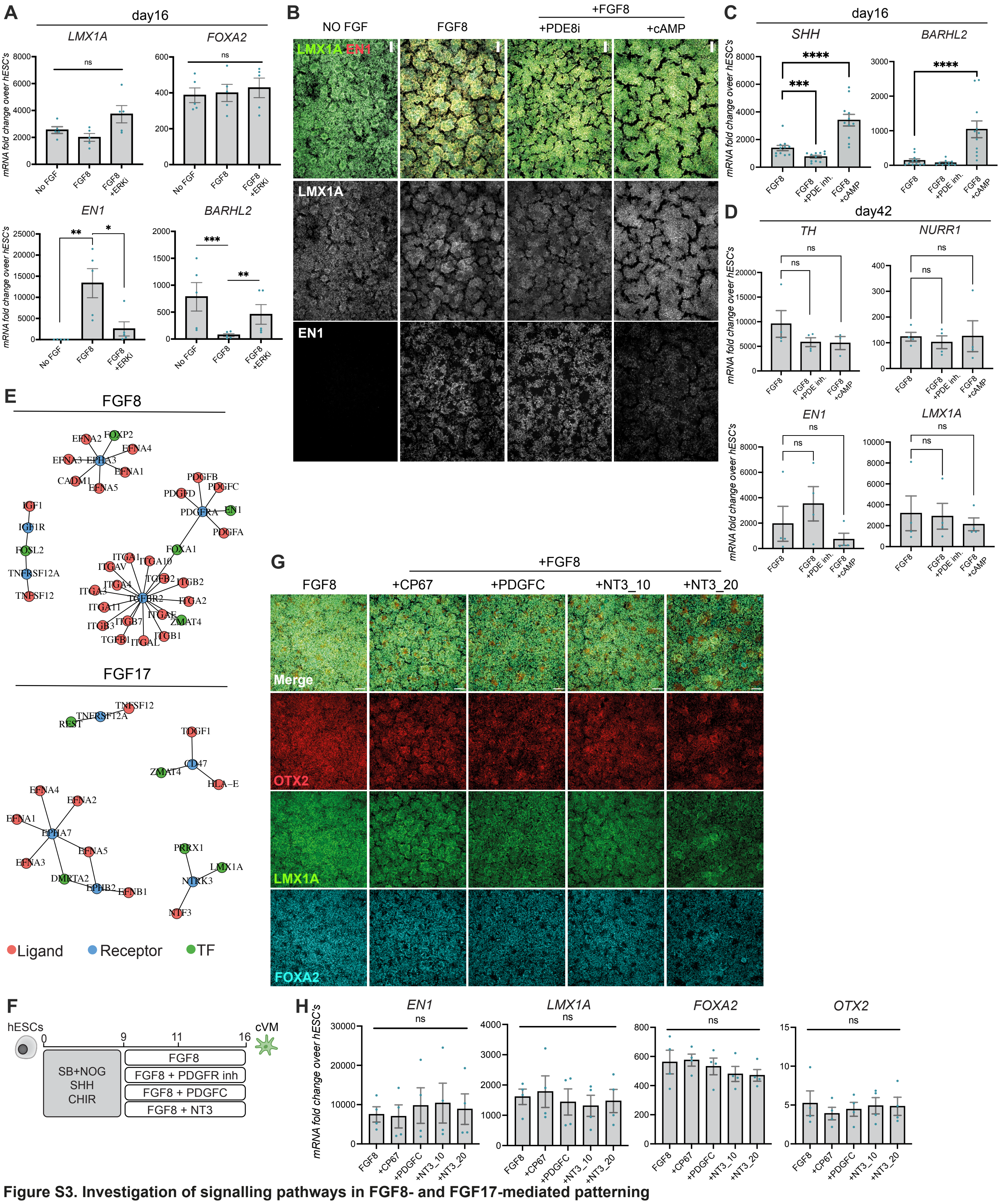

Figure S3. Investigation of signalling pathways in FGF8- and FGF17-mediated patterning
A. Quantitative RT-PCR of VM markers in day 16 progenitors treated with an ERK inhibitor (Trametinib) from day 9-16 in combination with FGF8. Difference in expression between groups were tested using One-way ANOVA for paired data followed by Dunnet's multipel comparisons test. *p<0.05, **p<0.01, ***p<0.001, ns: non-significant, n=5. **B.** ICC of day 16 VM DA cultures treated with either PDE8i (PF-04957325) or cAMP together with FGF8 from day 9-16, scalebar = 100μM. **C.** Quantitative RT-PCR of VM markers in d16 progenitors treated with FGF8+PDE8i or FGF8+cAMP d9-16. One-way ANOVA for paired data followed by Dunnet's multiple comparisons test, n=11 **D.** mRNA levels of mature DA neuron markers in day 42 cultures treated FGF8+PDEi or FGF8+cAMP day9-16. One-way ANOVA for paired data followed by Dunnet's multiple comparisons analysis, n=4. **E.** Intercellular signalling network of the "caudal VM progenitor" cluster from either FGF8-treated or FGF17-treated cultures in the day 16 scRNAseq data, using Domino. **F.** Schematic of testing conditions performed on the basis of Domino results. **G.** ICC of day 16 VM DA cultures treated with either PDGFR inhibitor (CP67), PDGFC or NT-3 in combination with FGF8 from day9-16, scalebar = 100μM. **H.** mRNA levels of key VM markers in day 16 progenitors treated with the same compounds as in F. One-way ANOVA for paired data followed by Dunnet's multiple comparisons test or Friedman test followed by Dunn's multiple comparison, ns: non-significant, n=4.

Supplementary tables

Table S1. List of DEGs from bulk RNAseq FGF8 vs FGF17 d9-10 (xlsx file)

Table including information on the gene name, baseMean, log2FoldChange, lfcSE, stat, pvalues, padj, sig and condition from the DEG analysis.

Table S2. List of DEGs from scRNAseq FGF8 vs FGF17 d16 (xlsx file)

Table including information on gene name, pval, avg_log2FC, pct.1, pct.2 and p_val_adj from DEG analysis. Positive avg_log2FC means enriched in FGF8, negative avg_log2FC means enriched in FGF17.

Table S3. List of reagents and the dilutions.

Reagent	Dilution	Manufacturer	Cat. #
ALDH1A1	1:1000	AbCam	ab52492
hNCAM	1:1000	Santa Cruz Biotechnology	Sc-106
TH	1:2000	Merck Millipore	AB152
TH	1:1000	Merck Millipore	AB1542
HuNu	1:1000	Merck Millipore	MAB1281
FOXA2	1:400	R&D Systems	AF2400-SP
FOXA2	1:1000	Santa Cruz Biotechnology	sc-101060
LMX1A	1:1000	Merck Millipore	AB10533
MAP2	1:1000	Sigma	M1046
OTX2	1:500	R&D Systems	AF1979
EN1	1:1000	Novo Nordisk A/S	N/A
Anti-Goat Cy3	1:200	Jackson ImmunoResearch	705-165-147
Anti-mouse 488	1:200	Jackson ImmunoResearch	715-545-150
Anti-Rabbit 647	1:200	Jackson ImmunoResearch	711-605-152
Horse anti-mouse	1:200	Vector Laboratories	BA-2001
Rabbit anti-sheep	1:200	Vector Laboratories	BA-6000
Goat anti-rabbit	1:200	Vector Laboratories	BP-9100
Haematoxylin Gill No. II	-	Sigma Aldrich	GHS232-1L

Table S4. List of primers.

Gene	Full name	Primer sequence (fw/rv)
ACTB	Beta-actin	CCTTGCACATGCCGGAG/ GCACAGAGCCTCGCCTT
BARHL1	BarH like homeobox 1	GTACCAGAACCGCAGGACTAAA/ AGAAATAAGGCGACGGGAACAT
BARHL2	BarH like homeobox 2	GGAGATTACGAGTAGCCGTGAG/ AAGCTACGCTCCAGTTGATTGA
CORIN	Corin, serine peptidase	CATATCTCCATCGCCTCAGTTG/

		GGCAGGAGTCCATGACTGT
EN1	Engrailed 1	CGTGGCTTACTCCCCATTTA/ TCTCGCTGTCTCTCCCTCTC
FOXA2	Forkhead box A2	CCGTTCTCCATCAACAACCT/ GGGGTAGTGCATCACCTGTT
FOXG1	Forkhead box G1 (BF1)	TGGCCCATGTCGCCCTTCCT/ GCCGACGTGGTGCCGTTGTA
GAPDH	Glyceraldehyde-3-phosphate dehydrogenase	TTGAGGTCAATGAAGGGGTC/ GAAGGTGAAGGTCGGAGTCA
HOXA2	Homeobox A2	CGTCGCTCGCTGAGTGCCTG/ TGTCGAGTGTGAAAGCGTCGAGG
LMX1A	LIM homeobox transcription factor a	CGCATCGTTTCTTCTCCTCT/ CAGACAGACTTGGGGCTCAC
NKX2-1	NK2 homeobox 1	AGGGCGGGGCACAGATTGGA/ GCTGGCAGAGTGTGCCCAGA
OTX2	Orthodenticle homeobox 1	ACAAGTGGCCAATTCACTCC/ GAGGTGGACAAGGGATCTGA
PAX6	Paired box 6	TGGTATTCTCTCCCCCTCCT/ TAAGGATGTTGAACGGGCAG
PITX2	Paired like homeodomain 2	AACTCTATGAACGTCAACCCCC/ CGACATGCTCATGGACGAGATA
SHH	Sonic hedgehog	CCAATTACAACCCCGACATC/ AGTTTCACTCCTGGCCACTG
TH	Tyrosine hydroxylase	CGGGCTTCTCGGACCAGGTGTA/ CTCCTCGGCGGTGTACTCCACA
WNT1	Wingless-type MMTV integration site family, member 1	GAGCCACGAGTTTGGATGTT/ TGCAGGGAGAAAGGAGAGAA
EGR4	Early growth response 4	ACAGCGGTAGCTTCTTCATTCA/ CTAAGATGCCCGACATGAGGTT
FOSB	FosB proto-oncogene, AP-1 transcription factor unit	GACCGTAGCTGAAGCTGATTCT/ TACCTCTTGAAAGTGCAGGCTC
